# Serum Amylase Levels in Relation to Islet β Cell Function in Patients with Early Type 2 Diabetes

**DOI:** 10.1371/journal.pone.0162204

**Published:** 2016-09-08

**Authors:** Lei Zhuang, Jian-bin Su, Xiu-lin Zhang, Hai-yan Huang, Li-hua Zhao, Feng Xu, Tong Chen, Xue-qin Wang, Gang Wu, Xiao-hua Wang

**Affiliations:** 1 Department of Endocrinology, The Second People's Hospital of Nantong City, No. 43 Xinglong Street, Gangzha district, Nantong, 226002, China; 2 Department of Endocrinology, The Second Affiliated Hospital of Nantong University, No. 6 North Hai-er-xiang Road, Nantong, 226001, China; 3 Department of Clinical Laboratory, The Second Affiliated Hospital of Nantong University, No. 6 North Hai-er-xiang Road, Nantong, 226001, China; University of Bremen, GERMANY

## Abstract

**Objective:**

The insulin-pancreatic acinar axis may play a major role in pancreatic function. Amylase is an exocrine enzyme that is produced by pancreatic acinar cells, and low serum amylase levels may be associated with endocrine diseases, such as metabolic syndrome and diabetes. We hypothesized that low serum amylase levels may be associated with impaired islet β cell function in type 2 diabetes. Therefore, we investigated the relationship between the serum amylase levels and islet β cell function in patients with early type 2 diabetes.

**Methods:**

The cross-sectional study recruited 2327 patients with a mean of 1.71±1.62 years since their diagnosis of type 2 diabetes, and all participants were treated with lifestyle intervention alone. Serum amylase levels, the 75-g oral glucose tolerance test (OGTT) and metabolic risk factors were examined in all participants. The insulin sensitivity index (Matsuda index, ISI_Matsuda_) and insulin secretion index (ratio of total area-under-the-insulin-curve to glucose-curve, AUC_ins/glu_) were derived from the OGTT. Integrated islet β cell function was assessed by the Insulin Secretion-Sensitivity Index-2 (ISSI-2) (ISI_Matsuda_ multiplied by AUC_ins/glu_).

**Results:**

Serum amylase levels in the normal range were significantly correlated with ISI_Matsuda_, AUC_ins/glu_ and ISSI-2 (*r* = 0.203, 0.246 and 0.413, respectively, *p*<0.001). The association of the serum amylase levels with ISSI-2 (adjusted *r* = 0.363, *p*<0.001) was closer than the association with ISI_Matsuda_ (adjusted *r* = 0.191, *p*<0.001) and AUC_ins/glu_ (adjusted *r* = 0.174, *p*<0.001) after adjusting for the anthropometric indices, time since the diagnosis of diabetes, lipid profiles, uric acid levels, estimated glomerular filtration rate, HbA1c levels, smoking and drinking using the partial correlation test. After adjusting for these metabolic risk factors in the multivariate regression analysis with the amylase levels as the dependent variable, ISSI-2 was the major independent contributor to the serum amylase levels (*β* = 0.416, *t* = 21.72, *p*<0.001). Meanwhile, in a comparison of the groups with the highest and lowest quartiles of serum amylase levels, the mean difference in logISSI-2 was 0.902 (95% CI 0.823 to 0.982), and after adjusting for metabolic risk factors, the mean difference in logISSI-2 was 0.610 (0.537 to 0.683).

**Conclusions:**

Serum amylase levels in the normal range are positively associated with integrated islet β cell function in patients with early type 2 diabetes, as assessed by ISSI-2.

## Introduction

Amylase is an exocrine enzyme that is produced by pancreatic acinar cells. The elevated serum amylase levels are widely used as screening test for acute pancreatitis in clinical practice [1,2]. Moreover, serum amylase levels are also elevated in other conditions, including diabetic ketoacidosis [3,4] and renal insufficiency [5,6]. Low serum amylase levels are observed in individuals with chronic pancreatitis [7].

Recent studies showed that the serum amylase levels may be associated with endocrine and metabolic diseases [8, 9]. Low serum amylase levels were associated with increased risks of metabolic abnormalities, metabolic syndrome and diabetes. A previous study by Muneyuki et al. [10] of asymptomatic middle-aged adults showed that low serum amylase levels were associated with decreased basal insulin levels and insulin secretion, as well as increased insulin resistance. However, the relationship between the serum amylase levels and islet β cell function in type 2 diabetes has not been fully elucidated.

Insulin secretion and insulin sensitivity could be quantified with the hyperglycemic and euglycemic insulin clamp techniques, respectively [11]. However, these techniques are so time-consuming and labor intensive that they are difficult to apply in clinical practices. Surrogate measures of insulin secretion and insulin sensitivity have been derived from the oral glucose tolerance test (OGTT). The insulin sensitivity index of Matsuda (ISI_Matsuda_) from the OGTT is a validated measure of whole body insulin sensitivity [12]. The ratio of total area-under-the-insulin-curve to glucose-curve (AUC_ins/glu_) is a reasonable estimate of insulin secretion [13]. The product of insulin secretion and sensitivity (ISI_Matsuda_ multiplied by AUC_ins/glu_), also termed the Insulin Secretion-Sensitivity Index-2 (ISSI-2), is a useful marker of integrated islet β cell function [13,14]. As a composite measure, ISSI-2 may be a better index to reflect islet β cell function than either ISI_Matsuda_ or AUC_ins/glu_ alone.

The present study we designed was to investigate the relationship between the serum amylase levels and islet β cell function in patients with type 2 diabetes, as assessed by ISSI-2.

## Methods

### Ethics statement

The study was approved by the Institutional Review Board of the Second Affiliated Hospital of Nantong University, and written informed consent was obtained from all participants.

### Study design and participants

This cross-sectional study was conducted in patients with type 2 diabetes at the Endocrinology Department of the Second Affiliated Hospital of Nantong University and the Second People's Hospital of Nantong from January 2011 to December 2015. The inclusion criteria were: diagnosis of type 2 diabetes based on the ADA diagnostic criteria 2011 [15], less than 7 years since the diagnosis of diabetes, current treatment with lifestyle intervention alone for more than 3 months, and serum amylase levels in the normal range of 10–150 U/L. The exclusion criteria were: current treatment with hypoglycemic agents, type 1 diabetes, testing positive for glutamic acid decarboxylase antibody or insulin antibody, diuretic use, chronic hepatic disease, chronic kidney disease, malignancy, excessive drinking (alcohol consumption of more than 40 g of ethanol daily for women and 60 g daily for men), acute complications, such as diabetic ketoacidosis, and other disorders affecting glucose metabolism, such as hyperthyroidism. **S1 STROBE Checklist** had provided the STROBE checklist for the cross-sectional study.

Body mass index (BMI), systolic blood pressure (SBP) and diastolic blood pressure (DBP) were measured in all participants. SBP≥140 mmHg, DBP≥90 mmHg, or patients who were receiving hypertensive treatment were considered hypertensive.

### OGTT procedures

As previously described in detail [16], the 75-g OGTT was performed in the early morning after an overnight fast. Blood samples were taken at 0, 30, 60, 90, and 120 min to measure the serum glucose and insulin levels (glucose unit: mmol/L, insulin unit: miu/L). The insulin sensitivity index was estimated by the ISI_Matsuda_: ISI_Matsuda_ = 10000/square root of (Ins0 × Glu0) × (mean glucose × mean insulin during OGTT) [12]. The insulin secretion index was calculated from the ratio of total area-under-the-insulin-curve to area-under-the-glucose-curve (AUC_ins/glu_) using the trapezoidal rule [13]. Integrated islet β cell function was assessed by the Insulin Secretion-Sensitivity Index-2 (ISSI-2; ISI_Matsuda_ multiplied by AUC_ins/glu_) [13,14].

### Laboratory examination

Blood samples for the biochemical tests were also obtained in the morning after an overnight fast. HbA1c was measured by high performance liquid chromatography (D-10 Testing Program, Bio-Rad). The serum insulin assay used a magnetic bead-based enzymatic spectrofluorometric immunoassay with an automatic enzyme immunoassay apparatus (AIA360, TOSOH). The serum glucose, total cholesterol (TC), triglyceride (TG), high-density lipoprotein cholesterol (HDLC), low-density lipoprotein cholesterol (LDLC), uric acid (UA) and serum creatinine (Scr) levels were measured with Hitachi Model 7600 Series Automatic Analyzer. The glomerular filtration rate (eGFR) was estimated using the Modification of Diet in Renal Disease (MDRD) Study equation (eGFR = 175×(standardized Scr) –^1.154^ ×age^–0.203^×0.742 [if female]) [17].

### Statistical analyses

Data analyses were performed using the SPSS 16.0 statistical software (SPSS Inc., USA). The total participants were divided into four subgroups according to the quartiles of the serum amylase levels. Continuous variables were expressed as the means ± standard deviation (SD) or medians (interquartile range) in the case of skewed distributions. Categorical variables were described as frequencies (percentages). Logarithmic transformations were applied in non-normally distributed variables for further analyses.

The one-way analysis of variance (ANOVA) test was applied to compare differences in the continuous variables between the quartiles of the serum amylase levels. The Chi-squared test was applied to compare categorical variables between the four groups. The correlation between the serum amylase levels and islet β cell function indices were analyzed with Pearson’s correlations and the partial correlation test.

Multiple stepwise linear regression analyses were conducted to investigate the association of ISSI-2 and other metabolic factors as the independent variables with the serum amylase levels as the dependent variable. Meanwhile, the mean differences (B[95% CI]) in ISSI-2 between the quartiles of the serum amylase levels were also investigated using a multivariate linear regression analysis. The lowest quartile of the serum amylase levels was used as a reference. *p*<0.05 was considered statistically significant.

## Results

### Clinical characteristics of the participants

**Fig 1:**
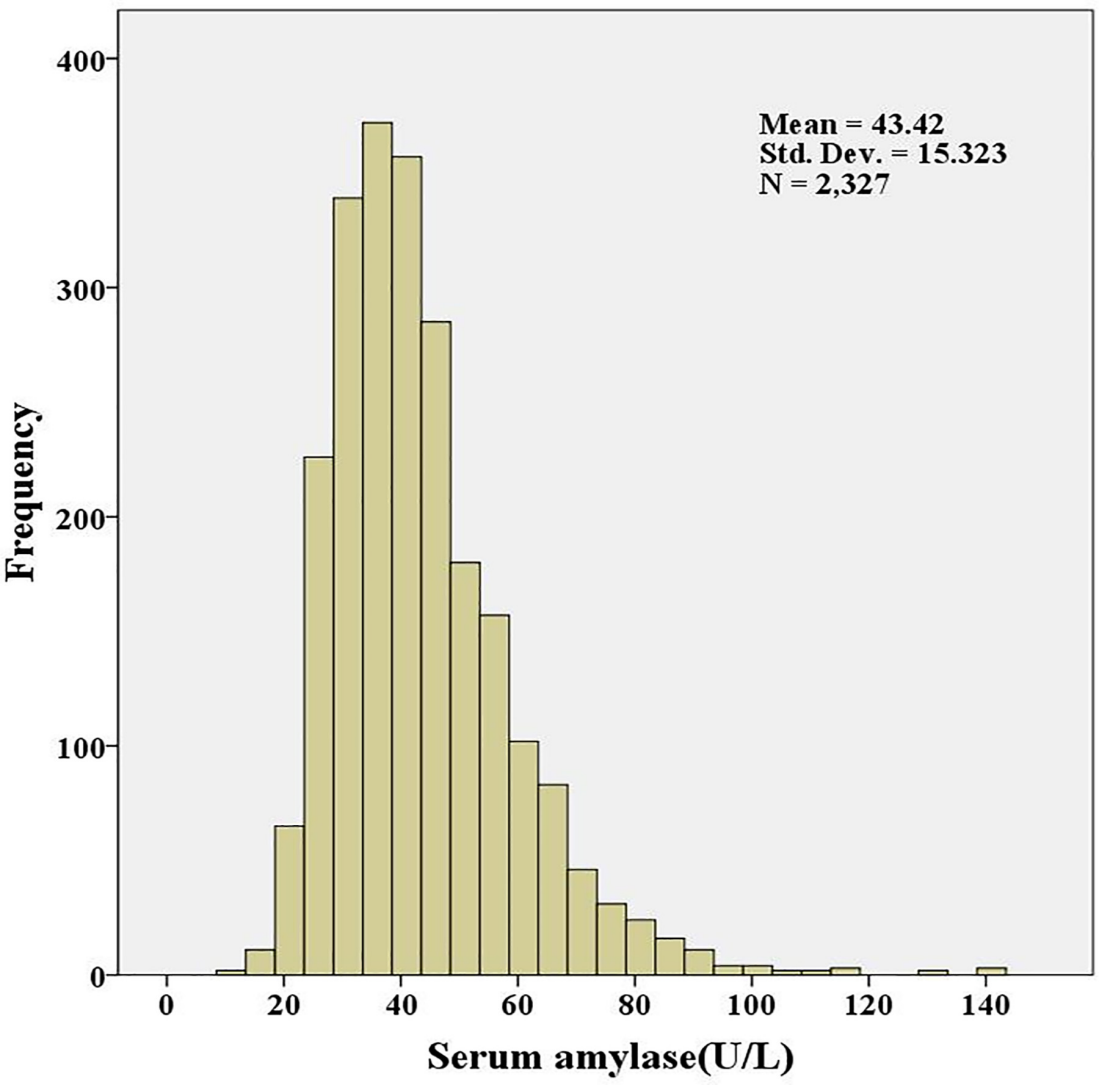
The distribution of the serum amylase levels.

A total of 2327 patients with a mean of 1.71±1.62 years since the diagnosis of type 2 diabetes were enrolled in the study, and all participants were treated with lifestyle intervention alone. The clinical characteristics of the participants, according to the serum amylase quartiles, were shown in **Table 1**, and the distribution of the serum amylase levels was shown in **Fig 1**. The mean serum amylase level among all the participants was 43.4±15.3 U/L. The quartiles of the serum amylase levels were 28.3±3.8 U/L, 37.1±2.0 U/L, 45.2±3.0 U/L and 64.8±13.5 U/L from the lowest to highest quartile, respectively. The age of the participants was 58.5±13.1 years, the percentage of females was 46.2% (n = 1074), BMI was 25.0±3.6 kg/m^2^, and the HbA1c levels were 8.22±1.19%. The prevalences of hypertension, smoking and moderate drinking in the participants were 25.5% (n = 594), 26.6% (n = 619) and 17.2% (n = 400), respectively. As the quartiles of the serum amylase levels increased, the female percentage, BMI, TG, eGFR and HbA1c levels were significantly decreased, whereas the age, Scr and UA levels were increased. The time since the diagnosis of diabetes, SBP, DBP, percentages of hypertension and moderate drinking, and TC, HDLC and LDLC levels did not show differences among the quartiles of the amylase levels, with the exception of the percentage of smoking compared to the amylase quartiles **(Table 1)**.

**Table 1:** Clinical characteristics of the participants according to the amylase quartiles.

Variables	Total	Q1	Q2	Q3	Q4	*p* value for the trend
Amylase, U/L (range)	43.4±15.3 (11–142)	28.3±3.8 (11–33)	37.1±2.0 (34–40)	45.2±3.0 (41–51)	64.8±13.5 (52–142)	<0.001
n	2327	643	524	599	561	–
Age (year)	56.1±13.0	54.5±13.2	55.5±12.1	55.8±13.3	58.5±13.1	<0.001
Female, n (%)	1074(46.2)	325(50.5)	262(50.0)	267(44.6)	220(39.2)	<0.001
BMI (kg/m^2^)	25.0±3.6	25.3±3.8	25.1±3.7	24.9±3.4	24.6±3.6	0.031
SBP (mmHg)	134±18	134±17	134±17	134±18	135±18	0.959
DBP (mmHg)	80±10	80±10	80±10	80±10	80±11	0.631
Time since the diagnosis of diabetes (years)	1.71±1.62	1.71±1.55	1.70±1.67	1.67±1.61	1.75±1.67	0.930
Hypertension, n (%)	594(25.5)	154(24.0)	233(25.4)	153(25.0)	154(27.5)	0.182
Smoking, n (%)	619(26.6)	198(30.8)	128(24.4)	160(26.7)	133(23.7)	0.015
Drinking, n (%)	400(17.2)	122(19.0)	88(16.8)	104(17.4)	86(15.3)	0.129
TG (mmol/L)	1.59 (1.02–2.51)	1.69 (1.05–2.80)	1.59 (1.03–2.54)	1.60 (1.02–2.40)	1.46 (0.98–2.30)	0.025
TC (mmol/L)	4.77±1.25	4.83±1.55	4.74±1.10	4.80±1.18	4.69±1.08	0.220
HDLC (mmol/L)	1.08±0.29	1.06±0.34	1.08±0.26	1.10±0.28	1.10±0.27	0.089
LDLC (mmol/L)	2.51±0.82	2.50±0.85	2.54±0.84	2.55±0.81	2.43±0.77	0.083
Scr (μmol/L)	57.8±21.2	53.5±16.4	54.8±15.5	57.8±19.5	65.3±29.1	<0.001
UA (μmol/L)	281±100	269±96	273±92	285±100	299±110	<0.001
eGFR (ml/min/1.73 m^2^)	123±36	131±38	126±33	122±35	111±36	<0.001
HbA1c (%)	8.22±1.19	8.67±1.07	8.33±1.16	8.08±1.14	7.76±1.20	<0.001

Normally distributed values in the table are presented as the means ± SD, non-normally distributed values are presented as medians (25% and 75% interquartiles), and categorical variables are presented as frequencies (percentages).

BMI: body mass index; SBP/DBP: systolic/diastolic blood pressure; TC: total cholesterol; TG: triglyceride; HDLC: High-density lipoprotein cholesterol; LDLC: Low-density lipoprotein cholesterol; UA: uric acid; Scr: serum creatinine; HbA1c: glycosylated hemoglobin A1c; eGFR: estimated glomerular filtration rate.

*p* values for the continuous variables and categorical variables were determined by ANOVA and the Chi-squared test, respectively.

### Serum glucose, insulin and islet β cell function indices from the OGTT

The statistical comparisons of the measurements characterizing the plasma glucose and insulin levels during the OGTT were summarized in **Table 2**. The serum glucose levels at baseline, 30, 60, 90 and 120 min were significantly decreased from the lowest to highest quartiles of the serum amylase levels (*p* value for the trend <0.001). There were no differences in the serum insulin levels at baseline among the four subgroups (*p* value for the trend = 0.190), whereas the insulin levels were significantly increased at 30, 60, 90 and 120 min from the lowest to highest quartiles of the serum amylase levels (*p* value for the trend <0.001). **S1–S3 Figs** showed the skewed distributions of the insulin sensitivity index (ISI_Matsuda_), insulin secretion index (AUC_ins/glu_) and integrated islet β cell function (ISSI-2). When these indices were log-transformed, logISI_Matsuda_, logAUC_ins/glu_ and logISSI-2 presented normal distributions (**S4–S6 Figs**). ISI_Matsuda_, AUC_ins/glu_ and ISSI-2 were significantly increased as the quartiles of the serum amylase levels increased (*p* value for the trend <0.001; **Table 2**).

**Table 2:** Serum glucose, insulin and islet β cell function indices from the OGTT according to the amylase quartiles.

Variables	Total	Q1	Q2	Q3	Q4	*p* value for the trend
Amylase, U/L (range)	43.4±15.3 (11–142)	28.3±3.8 (11–33)	37.1±2.0 (34–40)	45.2±3.0 (41–51)	64.8±13.5 (52–142)	<0.001
n	2327	643	524	599	561	–
Glu0 (mmol/L)	7.06±0.66	7.32±0.72	7.09±0.65	6.96±0.61	6.86±0.55	<0.001
Glu30 (mmol/L)	10.9±1.2	11.3±1.2	11.0±1.3	10.9±1.2	10.6±1.1	<0.001
Glu60 (mmol/L)	11.8±2.0	12.6±1.7	11.9±1.9	11.5±2.0	11.2±2.1	<0.001
Glu90 (mmol/L)	13.9±1.5	14.3±1.3	14.0±1.5	13.8±1.5	13.4±1.6	<0.001
Glu120 (mmol/L)	15.4±1.9	16.1±1.5	15.5±1.8	15.1±2.0	14.8±2.1	<0.001
Ins0 (miu/L)	7.4 (4.7–11.3)	7.7 (4.7–11.5)	7.3 (4.8–11.3)	7.6 (4.8–11.2)	7.1 (4.6–11.1)	0.190
Ins30 (miu/L)	14.6 (9.1–23.6)	12.2 (7.9–18.7)	14.5 (8.7–22.5)	15.5 (9.2–25.4)	17.9 (11.0–30.5)	<0.001
Ins60 (miu/L)	15.0 (9.3–24.4)	14.1 (8.8–22.0)	14.3 (9.3–23.6)	15.7 (9.5–25.4)	16.5 (10.2–28.5)	<0.001
Ins90 (miu/L)	20.0 (12.1–34.8)	16.0 (10.4–25.8)	19.8 (12.3–31.0)	21.3 (12.3–39.5)	26.5 (15.3–43.5)	<0.001
Ins120 (miu/L)	22.0 (13.2–38.2)	18.3 (10.8–29.2)	20.9 (12.8–35.8)	23.5 (13.8–43.5)	27.5 (16.0–48.3)	<0.001
ISI_Matsuda_	133 (100–181)	117 (85–167)	126 (96–177)	131 (104–182)	153 (117–201)	<0.001
logISI_Matsuda_	4.93±0.49	4.82±0.54	4.89±0.47	4.95±0.48	5.07±0.42	<0.001
AUC_ins/glu_	2.58 (1.45–4.76)	1.99 (1.20–3.22)	2.42 (1.43–4.22)	2.82 (1.56–5.65)	3.55 (1.98–6.83)	<0.001
logAUC_ins/glu_	1.00±0.91	0.68±0.79	0.93±0.85	1.11±0.93	1.33±0.96	<0.001
ISSI-2	329 (214–601)	233 (168–333)	313 (210–494)	382 (253–690)	553 (315–1041)	<0.001
logISSI-2	5.92±0.78	5.49±0.57	5.81±0.67	6.07±0.75	6.39±0.83	<0.001

Normally distributed values in the table are presented as the means ± SD, non-normally distributed values are presented as medians (25% and 75% interquartiles).

Glu*t*: plasma glucose levels at time *t* during the OGTT; Ins*t*: plasma insulin levels at time *t* during the OGTT; ISI_Matsuda_: insulin sensitivity index; AUC_ins/glu_: ratio of total area-under-the-insulin-curve to area-under-the-glucose-curve; ISSI-2: Insulin Secretion-Sensitivity Index-2.

*p* values for the continuous variables and categorical variables were determined by ANOVA and the Chi-squared test, respectively.

### Relationships between the serum amylase levels, β cell function indices and metabolic risk factors

**Fig 2:**
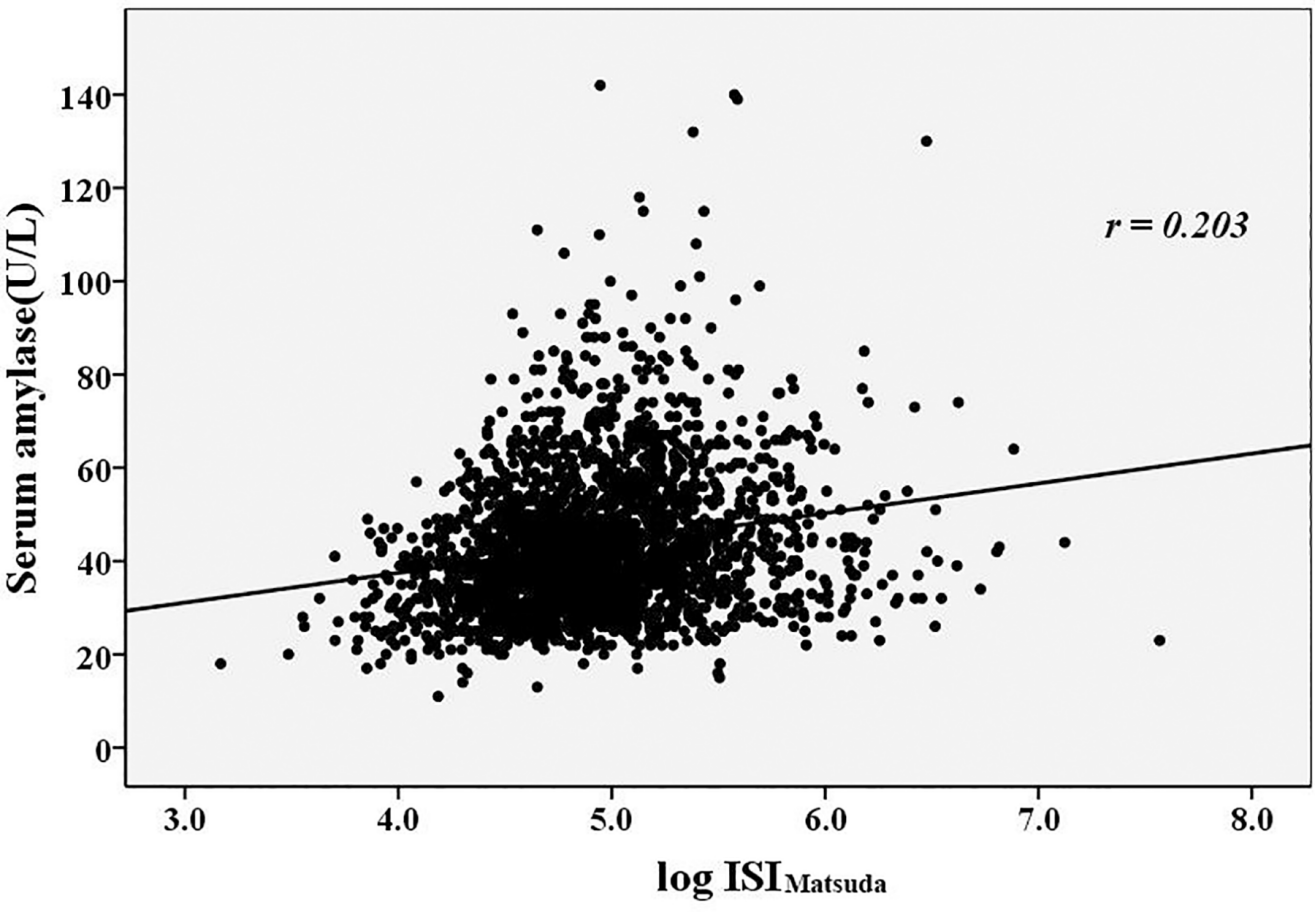
The relationship between the serum amylase levels and ISI_Matsuda_. ISI_Matsuda_: insulin sensitivity index of Matsuda.

**Fig 3:**
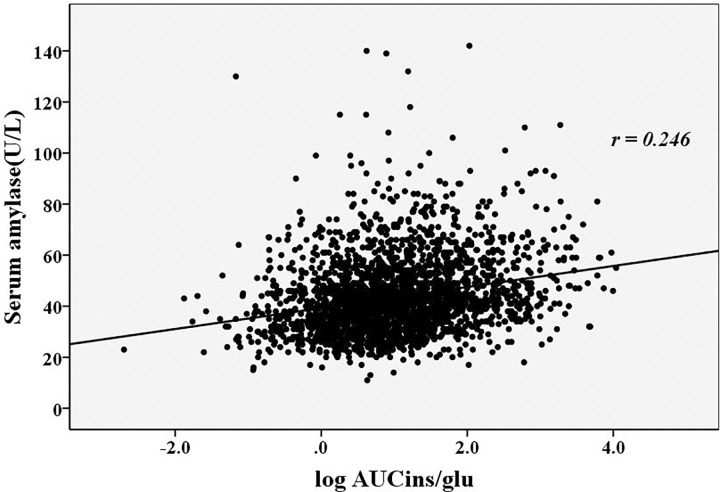
The relationship between the serum amylase levels and AUC_ins/glu_. AUC_ins/glu_: ratio of total area-under-the-insulin-curve to area-under-the-glucose-curve.

**Fig 4:**
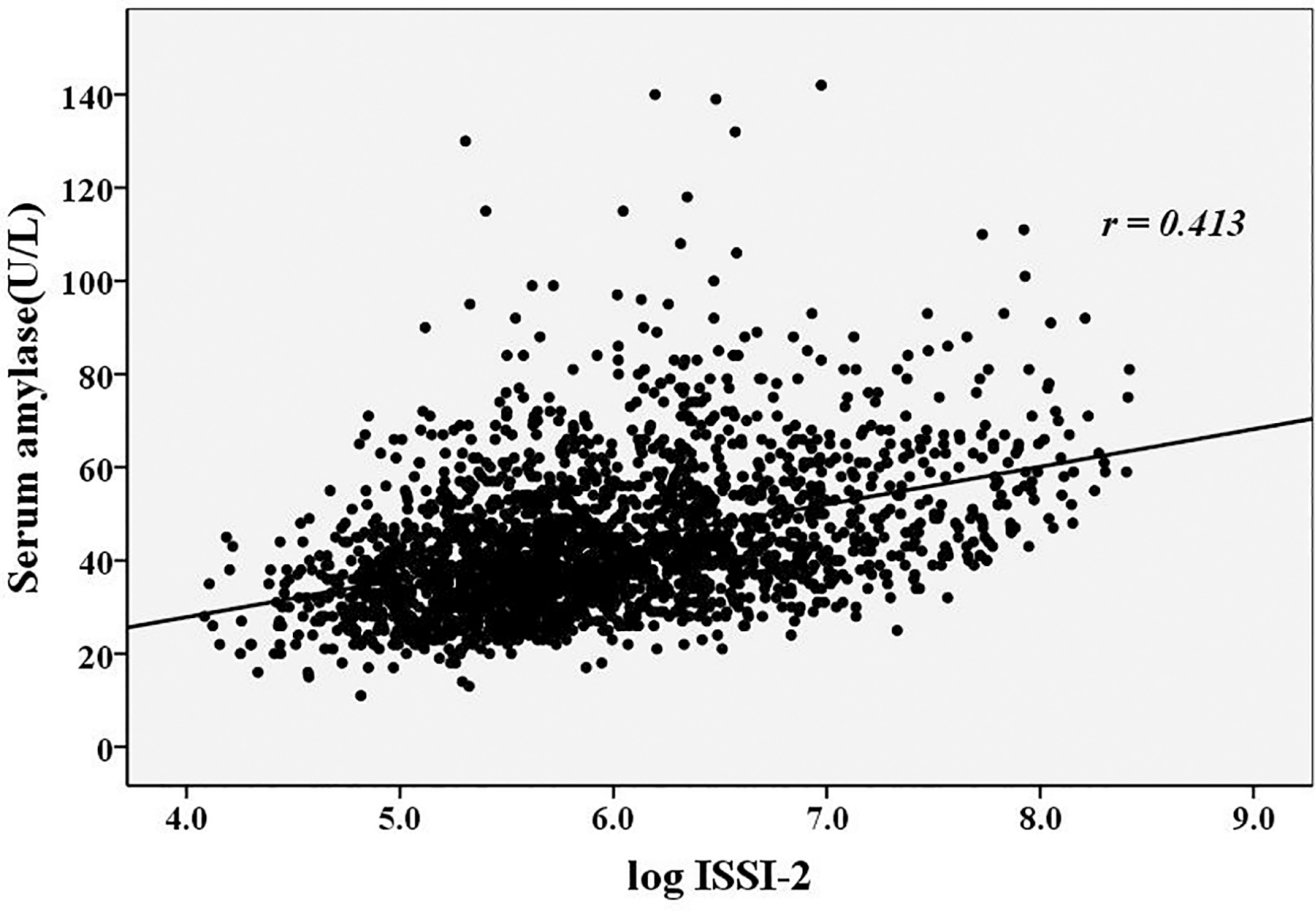
The relationship between the serum amylase levels and ISSI-2. ISSI-2: Insulin Secretion-Sensitivity Index-2.

**Table 3** showed the correlations between the serum amylase levels, β cell function indices and metabolic risk factors. Serum amylase levels in the normal range were positively correlated with age and the UA levels (*r* = 0.098, and 0.125, respectively, *p*<0.05) and were negatively correlated with BMI, TG levels, HbA1c levels, and eGFR (*r* = –0.077, –0.073, –0.267 and –0.214, respectively, *p*<0.05). The correlations between the serum amylase levels and the β cell function indices were presented in **Figs 2–4**. The serum amylase levels were significantly associated with ISI_Matsuda_, AUC_ins/glu_ and ISSI-2 (*r* = 0.203, 0.246 and 0.413, respectively, *p*<0.001; **Figs 2–4)**. After adjusting for the metabolic risks using the partial correlation test, the serum amylase levels remained associated with ISI_Matsuda_, AUC_ins/glu_ and ISSI-2 (adjusted *r* = 0.191, 0.174 and 0.363, respectively, *p*<0.001). The association of the serum amylase levels with ISSI-2 was closer than the association with ISI_Matsuda_ and AUC_ins/glu_.

**Table 3:** Correlations between the serum amylase levels, β cell function indices and metabolic risk factors.

Variables		Age	Time since the diagnosis of diabetes	BMI	SBP	DBP	TG	TC	HDLC	LDLC	eGFR	UA	HbA1c
Amylase	*r*	0.098	–0.006	–0.077	0.008	–0.030	–0.073	–0.046	0.059	–0.024	–0.214	0.125	–0.267
	*p*	<0.001	0.787	<0.001	0.712	0.144	<0.001	0.027	0.005	0.258	<0.001	<0.001	<0.001
logISI_Matsuda_	*r*	–0.012	–0.006	–0.246	–0.129	–0.067	–0.201	–0.091	0.134	0.015	0.023	–0.110	–0.126
	*p*	0.571	0.775	<0.001	<0.001	0.001	<0.001	<0.001	<0.001	0.472	0.261	<0.001	<0.001
logAUC_insglu_	*r*	–0.022	–0.189	0.238	0.067	0.025	0.093	–0.015	–0.075	–0.062	–0.125	0.238	–0.579
	*p*	0.284	<0.001	<0.001	0.001	0.237	<0.001	0.466	<0.001	0.003	<0.001	<0.001	<0.001
logISSI2	*r*	–0.033	–0.224	0.123	–0.002	–0.013	–0.017	–0.074	–0.003	–0.063	–0.131	0.209	–0.642
	*p*	0.109	<0.001	<0.001	0.936	0.538	0.419	<0.001	0.872	0.003	<0.001	<0.001	<0.001

BMI: body mass index; SBP/DBP: systolic/diastolic blood pressure; TC: total cholesterol; TG: triglyceride; HDLC: high density lipoprotein cholesterol; LDLC: low density lipoprotein cholesterol; UA: uric acid; HbA1c: glycosylated hemoglobin A1c; eGFR: estimated glomerular filtration rate; ISI_Matsuda_: insulin sensitivity index; AUC_ins/glu_: ratio of total area-under-the-insulin-curve to area-under-the-glucose-curve; ISSI-2: Insulin Secretion-Sensitivity Index-2

### Multivariate linear stepwise regression analysis with the amylase levels as the dependent variable

The association of the serum amylase levels with ISSI-2 was closer than the association with ISI_Matsuda_ and AUC_ins/glu_; therefore, a multivariate linear stepwise regression analysis was further performed to assess the association of ISSI-2 and other clinical risk factors as the independent variables with serum amylase levels as the dependent variable in the participants. These independent factors included age, sex (female), BMI, SDP, DBP, hypertension (no = 0, yes = 1), moderate drinking (no = 0, yes = 1), smoking (no = 0, yes = 1), time since the diagnosis of diabetes, TG, TC, HDLC, LDLC, UA, eGFR, HbA1c and ISSI-2. ISSI-2 was the major independent contributor to the serum amylase levels (standardized coefficient *β* = 0.416, *t* = 21.72, *p*<0.001, partial *R*^*2*^ = 16.5) (**Table 4**).

**Table 4:** Multiple linear regression analysis with the serum amylase levels in the participants as the dependent variables.

Variables	B	SE	*β*	*t*	*partial R*^*2*^(%)	*p*
logISSI-2	8.11	0.38	0.416	21.72	16.5	<0.001
eGFR	–0.067	0.008	–0.16	–8.40	2.7	<0.001
BMI	–0.48	0.074	–0.12	–6.49	1.7	<0.001
Female	–3.90	0.58	–0.13	–6.73	1.4	<0.001
Smoking	–2.89	0.66	–0.083	–4.31	0.5	<0.001

B, Regression coefficient; SE, Standard error; *β*, Standardized coefficient

### Multivariate linear regression analysis to investigate the mean differences in ISSI-2 between the quartiles of the amylase levels

Meanwhile, a multivariate linear regression analysis was also applied to investigate the differences in ISSI-2 between the quartiles of the amylase levels. By comparing the groups with the highest (Q4) and lowest (Q1) quartiles of serum amylase levels, the mean difference in log-transformed ISSI-2 was 0.902 (95% CI 0.823 to 0.982), and after adjusting for anthropometric indices and metabolic risk factors, the adjusted mean difference in log-transformed ISSI-2 was 0.610 (0.537 to 0.683) **(Table 5)**.

**Table 5:** Mean differences in logISSI-2 between the quartiles of the serum amylase levels [B (95% CI)].

Model	Q1–reference	Q2	Q3	Q4	*p* value for the trend
0	0	0.320(0.248, 0.391)	0.573(0.499, 0.647)	0.902(0.823, 0.982)	<0.001
1	0	0.316(0.246, 0.387)	0.575(0.503, 0.648)	0.942(0.861, 1.022)	<0.001
2	0	0.326(0.255, 0.396)	0.578(0.505, 0.650)	0.946(0.866, 1.026)	<0.001
3	0	0.225(0.163, 0.288)	0.389(0.323, 0.454)	0.610(0.537, 0.683)	<0.001

Model 0: unadjusted model.

Model 1: adjusted for age, sex, BMI, SBP and DBP.

Model 2: additionally adjusted for hypertension, moderate drinking, and smoking.

Model 3: additionally adjusted for time since the diagnosis of diabetes, HbA1c, UA, eGFR, TG, TC, HDLC and LDLC.

## Discussion

In the present study, we investigated the association of the serum amylase levels with insulin secretion, insulin sensitivity and integrated islet β cell function derived from the OGTT in a large Chinese population with early type 2 diabetes. Insulin secretion, insulin sensitivity and integrated islet β cell function were measured by the ISI_Matsuda_, AUC_ins/glu_ and ISSI-2, respectively. The strengths of our study showed that the serum amylase levels in a normal range were positively associated with ISSI-2, ISI_Matsuda_ and AUC_ins/glu_, but the association of the serum amylase levels with ISSI-2 was closer than the association with ISI_Matsuda_ and AUC_ins/glu_. The relationship between the serum amylase levels and ISSI-2 remained significant, even after adjusting for multiple metabolic risks in the multivariate regression analysis.

### The association of the serum amylase levels with insulin sensitivity and insulin secretion

Previous studies in animals had provided data indicating there was an insulin-pancreatic acinar axis that played a major role in pancreatic function [18–20]. These studies showed that low serum insulin levels were accompanied by a reduction in the serum amylase levels, and this reduction was reversed after insulin injections in the diabetic rats. Moreover, a recent clinical study supported the close relationship between the endocrine and exocrine pancreas. A community-based study by Nakajima et al. [21] showed that low serum amylase levels may reflect an increased risk of metabolic abnormalities and abnormal glucose metabolism, which were associated with insulin resistance and impaired insulin secretion. In a cross-sectional study of asymptomatic middle-aged adults by Muneyuki et al. [10], low serum amylase levels were associated with decreased basal insulin levels and insulin secretion. The results of our study showed that the serum amylase levels were positively associated with insulin secretion, as measured by AUC_ins/glu_, and insulin sensitivity, as measured by ISI_Matsuda_, in type 2 diabetic patients, even after adjusting for anthropometric indices and metabolic risk factors. Our study of a large population with type 2 diabetes was consistent with previous studies in asymptomatic subjects [10, 21]. Therefore, the serum amylase levels were positively associated with insulin secretion and insulin sensitivity in both asymptomatic subjects and type 2 diabetic patients.

### The association of the serum amylase levels with integrated islet β cell function

The serum amylase levels may also be a potential biomarker that is closely connected to overall islet β cell function. The observed association of the serum amylase levels in the normal range with ISSI-2 was closer than the association with ISI_Matsuda_ and AUC_ins/glu_ in our study. As recommended by Retnakaran et al. [13,14], ISSI-2 was shown to be a potential OGTT-based measure of β cell function. ISSI-2 was the major independent contributor to the serum amylase levels after the multivariate linear stepwise regression analysis. Meanwhile, the mean differences in ISSI-2 were significant when comparing groups with different quartiles of amylase levels, even after adjusting for other metabolic risk factors. The study of Muneyuki et al. [10] showed that low serum amylase levels were associated with the plasma insulin levels at 0 and 60 min during the OGTT, indicating that low serum amylase levels may be associated with low insulin action in the basal status and for up to 60 min after glucose loading. The strength of our study showed that the serum amylase levels were closely associated with ISSI-2, which contained whole-body insulin sensitivity and total insulin secretion at all times (120 min) after glucose loading. Further study is needed to investigate the role of the improvement of ISSI-2 in the changes in the serum amylase levels and determine whether there is a causal relationship between the serum amylase levels and ISSI-2.

### The relationship between the amylase levels and other metabolic risk factors

Serum amylase levels may relate to other metabolic risk factors that are associated with impaired insulin secretion and insulin action. The results from previous studies suggested that low serum amylase levels were significantly associated with BMI, blood pressure, γ-glutamyltransferase levels, TG levels, fasting plasma glucose levels, serum adiponectin levels, non-alcoholic fatty liver disease and metabolic syndrome [22–24]. HbA1c, which determines the overall glycemic levels by integrating both fasting and postprandial hyperglycemia [25], was not reported to associate with the serum amylase levels [10]. However, in the present study, HbA1c was negatively associated with the serum amylase levels (*r* = –0.267) (**Table 3**). After the multivariate regression analysis, HbA1c was not an independent contributor to the serum amylase levels, possibly because HbA1c was closely related to ISSI-2 (*r* = –0.642). Hyperglycemia may exert a toxic effect on acinar amylase secretion and cause an apparent diminution of the exocrine reserves, suggesting an association between low circulating amylase levels and poor glycemic control [26]. Our study also indicated that sex, BMI, eGFR, and smoking were also independent contributors to the serum amylase levels. Smoking was risk for type 2 diabetes [27] and may latently correlate with the serum amylase levels. These findings enhanced the strengths of this study by showing that the serum amylase levels were closely associated with endocrine and metabolic diseases.

### The possible mechanisms by which insulin regulates amylase production

The basal mechanisms by which insulin regulates amylase production by pancreatic acinar cells had been addressed in previous studies [28–30]. Insulin acts by binding to its own receptor on acinar cells, leading to the stimulation and potentiation of amylase secretion through multiple signaling pathways, including regulation of amylase gene transcription and stimulation of the synthesis of the corresponding protein in acinar cells [31]. Patel et al. [30] showed that insulin could stimulate amylase release from acinar cells in healthy and diabetic rats, but with a much-reduced effect in diabetic rats. Moreover, studies in patients with diabetes mellitus showed pancreatic exocrine tissue fibrosis and a reduced response to hormonal stimulation [31,32]. Our present study showed that the low serum amylase levels were associated with impaired insulin secretion and insulin action in type 2 diabetes. The pathophysiological mechanisms of type 2 diabetes were a deficiency in both islet β cell secretion and impaired insulin action [33], which in turn may be responsible for decreasing basal serum amylase secretion.

### Limitations

It should be noted that our study has some limitations. First, it is a cross-sectional observational study that cannot definitively comment on the causality of the associations between the serum amylase levels and islet β cell function. Prospective longitudinal studies are needed to evaluate the cause-effect relationship. Second, the cross-sectional study was performed in a Chinese population, and our findings may lack generalizability to other populations. Third, the circulating insulin levels during the OGTT may be affected by other factors in addition to β cell function, such as incretin hormones and hepatic extraction. These two factors may limit the degree to which the insulin levels during the OGTT can reflect β cell function. Fourth, serum amylase can be classified as a pancreatic-type and salivary-type amylase, and our study does not distinguish between the two types for clinical relevance analysis. However, to our knowledge, few studies distinguish between the two types of amylase for further clinical analysis.

### Conclusions

In summary, the serum amylase levels in the normal range are positively associated with integrated islet β cell function in patients with early type 2 diabetes, as assessed by ISSI-2, even after adjusting for anthropometric indices and other metabolic risk factors. Serum amylase levels may be a potential biomarker that is closely connected to overall islet β cell function.

## Supporting Information

S1 STROBE Checklist The STROBE Checklist for the cross-sectional study. STROBE: STrengthening the Reporting of OBservational studies in Epidemiology.
(DOCX)

S1 Fig The distribution of ISI_Matsuda_. ISI_Matsuda_: insulin sensitivity index of Matsuda.
(TIF)

S2 Fig The distribution of AUC_ins/glu_. AUC_ins/glu_: ratio of total area-under-the-insulin-curve to area-under-the-glucose-curve.
(TIF)

S3 Fig The distribution of ISSI-2. ISSI-2: Insulin Secretion-Sensitivity Index-2.
(TIF)

S4 Fig The distribution of logISI_Matsuda_. ISI_Matsuda_: insulin sensitivity index of Matsuda.
(TIF)

S5 Fig The distribution of logAUC_ins/glu_. AUC_ins/glu_: ratio of total area-under-the-insulin-curve to area-under-the-glucose-curve.
(TIF)

S6 Fig The distribution of logISSI-2. ISSI-2: Insulin Secretion-Sensitivity Index-2.
(TIF)

## References

[1] Mäkelä A, Kuusi T, Schröder T. Serum phospholipase A2, amylase, lipase, and urinary amylase activities in relation to the severity of acute pancreatitis. Eur J Surg. 1997; 163: 915–922. 9449444

[2] Yadav D, Agarwal N, Pitchumoni CS. A critical evaluation of laboratory tests in acute pancreatitis. Am J Gastroenterol. 2002; 97: 1309–1318. 10.1111/j.1572-0241.2002.05766.x 12094843

[3] Quiros JA, Marcin JP, Kuppermann N, Nasrollahzadeh F, Rewers A, DiCarlo J, et al Elevated serum amylase and lipase in pediatric diabetic ketoacidosis. Pediatr Crit Care Med. 2008; 9: 418–422. 10.1097/PCC.0b013e318172e99b 18496406

[4] Rizvi AA. Serum amylase and lipase in diabetic ketoacidosis. Diabetes Care. 2003; 26: 3193–3194. 10.2337/diacare.26.11.3193 14578269

[5] Seno T, Harada H, Ochi K, Tanaka J, Matsumoto S, Choudhury R, et al Serum levels of six pancreatic enzymes as related to the degree of renal dysfunction. Am J Gastroenterol. 1995; 90: 2002–2005. 7485010

[6] Jiang CF, Ng KW, Tan SW, Wu CS, Chen HC, Liang CT, et al Serum level of amylase and lipase in various stages of chronic renal insufficiency. Zhonghua Yi Xue Za Zhi(Taipei). 2002; 65: 49–54. 12014357

[7] Gupta V, Toskes PP. Diagnosis and management of chronic pancreatitis. Postgrad Med J. 2005; 81: 491–497. 10.1136/pgmj.2003.009761 16085738 PMC1743323

[8] Nakajima K, Muneyuki T, Munakata H, Kakei M. Revisiting the cardiometabolic relevance of serum amylase. BMC Res Notes. 2001; 4: 419 10.1186/1756-0500-4-419 22004561 PMC3208003

[9] Lee JG, Park SW, Cho BM, Lee S, Kim YJ, Jeong DW, et al Serum amylase and risk of the metabolic syndrome in Korean adults. Clin Chim Acta. 2011; 412: 1848–1853. 10.1016/j.cca.2011.06.023 21726545

[10] Muneyuki T, Nakajima K, Aoki A, Yoshida M, Fuchigami H, Munakata H, et al Latent associations of low serum amylase with decreased plasma insulin levels and insulin resistance in asymptomatic middle-aged adults. Cardiovasc Diabetol. 2012; 11: 80 10.1186/1475-2840-11-80 22748134 PMC3439247

[11] Abdul-Ghani MA, Williams K, DeFronzo RA, Stern M. What is the best predictor of future type 2 diabetes? Diabetes Care. 2007; 30: 1544–1548. 10.2337/dc06-1331 17384342

[12] Matsuda M, DeFronzo RA. Insulin sensitivity indices obtained from oral glucose tolerance testing: comparison with the euglycemic insulin clamp. Diabetes Care. 1999; 22: 1462–1470. 10.2337/diacare.22.9.1462 10480510

[13] Retnakaran R, Shen S, Hanley AJ, Vuksan V, Hamilton JK, Zinman B. Hyperbolic relationship between insulin secretion and sensitivity on oral glucose tolerance test. Obesity (Silver Spring). 2008; 16: 1901–1907. 10.1038/oby.2008.307 18551118

[14] Retnakaran R, Qi Y, Goran MI, Hamilton JK. Evaluation of proposed oral disposition index measures in relation to the actual disposition index. Diabet Med. 2009; 26: 1198–1203. 10.1111/j.1464-5491.2009.02841.x 20002470

[15] American Diabetes Association. Diagnosis and classification of diabetes mellitus. Diabetes Care. 2011; 34: S62–69. 10.2337/dc11-S062 21193628 PMC3006051

[16] Wang YM, Zhao LH, Su JB, Qiao HF, Wang XH, Xu F, et al Glycemic variability in normal glucose tolerance women with the previous gestational diabetes mellitus. Diabetol Metab Syndr. 2015; 7: 82 10.1186/s13098-015-0077-5 26405461 PMC4581077

[17] Levey AS, Coresh J, Greene T, Stevens LA, Zhang YL, Hendriksen S, et al Using standardized serum creatinine values in the modification of diet in renal disease study equation for estimating glomerular filtration rate. Ann Intern Med. 2006; 145: 247–254. 16908915 10.7326/0003-4819-145-4-200608150-00004

[18] Aughsteen AA, Mohammed FI. Insulin enhances amylase and lipase activity in the pancreas of streptozotocin-diabetic rats. An in vivo study. Saudi Med J. 2002; 23: 838–844. 12174237

[19] Patel R, Yago MD, Mañas M, Victoria EM, Shervington A, Singh J. Mechanism of exocrine pancreatic insufficiency in streptozotocin-induced diabetes mellitus in rat: effect of cholecystokinin-octapeptide. Mol Cell Biochem. 2004; 261: 83–89. 10.1023/B:MCBI.0000028741.85353.c6 15362489

[20] Patel R, Shervington A, Pariente JA, Martinez-Burgos MA, Salido GM, Adeghate E, et al Mechanism of exocrine pancreatic insufficiency in streptozotocin-induced type 1 diabetes mellitus. Ann N Y Acad Sci. 2006; 1084: 71–88. 10.1196/annals.1372.038 17151294

[21] Nakajima K, Nemoto T, Muneyuki T, Kakei M, Fuchigami H, Munakata H, et al Low serum amylase in association with metabolic syndrome and diabetes: A community-based study. Cardiovasc Diabetol. 2011; 10: 34 10.1186/1475-2840-10-34 21496338 PMC3102610

[22] Eleftheriou P, Tseka E, Varaga E, Nasiou M, Sampanis C, Zografou I, et al Study of the lipidemic profile of diabetic patients. Negative correlation of cholesterol levels of diabetes type I patients with serum amylase concentration. Hell J Nucl Med. 2014; 17: S35–59. 24392467

[23] Zhao Y, Zhang J, Zhang J, Wu J, Chen Y. Metabolic syndrome and diabetes are associated with low serum amylase in a Chinese asymptomatic population. Scand J Clin Lab Invest. 2014; 74: 235–239. 10.3109/00365513.2013.878469 24456421

[24] Yao J, Zhao Y, Zhang J, Hong Y, Lu H, Wu J. Serum amylase levels are decreased in Chinese non-alcoholic fatty liver disease patients. Lipids Health Dis. 2014; 13: 185 10.1186/1476-511X-13-185 25481429 PMC4267431

[25] Nathan DM, Turgeon H, Regan S. Relationship between glycated haemoglobin levels and mean glucose levels over time. Diabetologia. 2007; 50: 2239–2244. 10.1007/s00125-007-0803-0 17851648 PMC8752566

[26] Zini E, Osto M, Moretti S, Franchini M, Kook PH, et al Hyperglycaemia but not hyperlipidaemia decreases serum amylase and increases neutrophils in the exocrine pancreas of cats. Res Vet Sci. 2010; 89: 20–26. 10.1016/j.rvsc.2010.01.006 20132955

[27] Yeh HC, Duncan BB, Schmidt MI, Wang NY, Brancati FL. Smoking, smoking cessation, and risk for type 2 diabetes mellitus: a cohort study. Ann Intern Med. 2010; 152: 10–17. 10.7326/0003-4819-152-1-201001050-00005 20048267 PMC5726255

[28] Lee KY, Lee YL, Kim CD, Chang TM, Chey WY. Mechanism of action of insulin on pancreatic exocrine secretion in perfused rat pancreas. Am J Physiol Gastrointest Liver Physiol. 1994; 267: G207–212. 7915495 10.1152/ajpgi.1994.267.2.G207

[29] Lee KY, Krusch D, Zhou L, Song Y, Chang TM, Chey WY. Effect of endogenous insulin on pancreatic exocrine secretion in perfused dog pancreas. Pancreas. 1995; 11: 190–195. 7479678 10.1097/00006676-199508000-00013

[30] Patel R, Pariente JA, Martinez MA, Salido GM, Singh J. Effect of insulin on acetylcholine-evoked amylase release and calcium mobilization in streptozotocin-induced diabetic rat pancreatic acinar cells. Ann N Y Acad Sci. 2006; 1084: 58–70. 10.1196/annals.1372.027 17151293

[31] Barreto SG, Carati CJ, Toouli J, Saccone GT. The islet-acinar axis of the pancreas: more than just insulin. Am J Physiol Gastrointest Liver Physiol. 2010; 299: G10–22. 10.1152/ajpgi.00077.2010 20395539

[32] Swislocki A, Noth R, Hallstone A, Kyger E, Triadafilopoulos G. Secretin-stimulated amylase release into blood is impaired in type 1 diabetes mellitus. Horm Metab Res. 2005; 37: 326–330. 10.1055/s-2005-861478 15971157

[33] Abdul-Ghani MA, Tripathy D, DeFronzo RA. Contributions of beta-cell dysfunction and insulin resistance to the pathogenesis of impaired glucose tolerance and impaired fasting glucose. Diabetes Care. 2006; 29: 1130–1139. 10.2337/diacare.2951130 16644654

