# Supplementary figures and images for: Serum Amylase Levels in Relation to Islet β Cell Function in Patients with Early Type 2 Diabetes
Source: PLoS One. 2016 Sep 8;11(9):e0162204. doi: 10.1371/journal.pone.0162204 (PMC5015989; doi:10.1371/journal.pone.0162204)

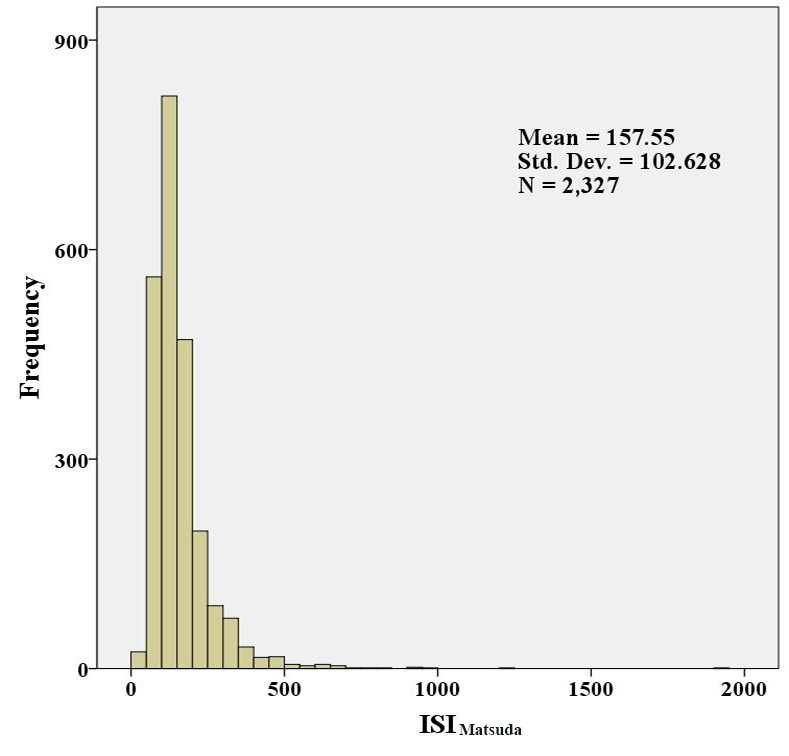

Supplement: S1 Fig — ISIMatsuda: insulin sensitivity index of Matsuda. (TIF) [file pone.0162204.s002.tif]

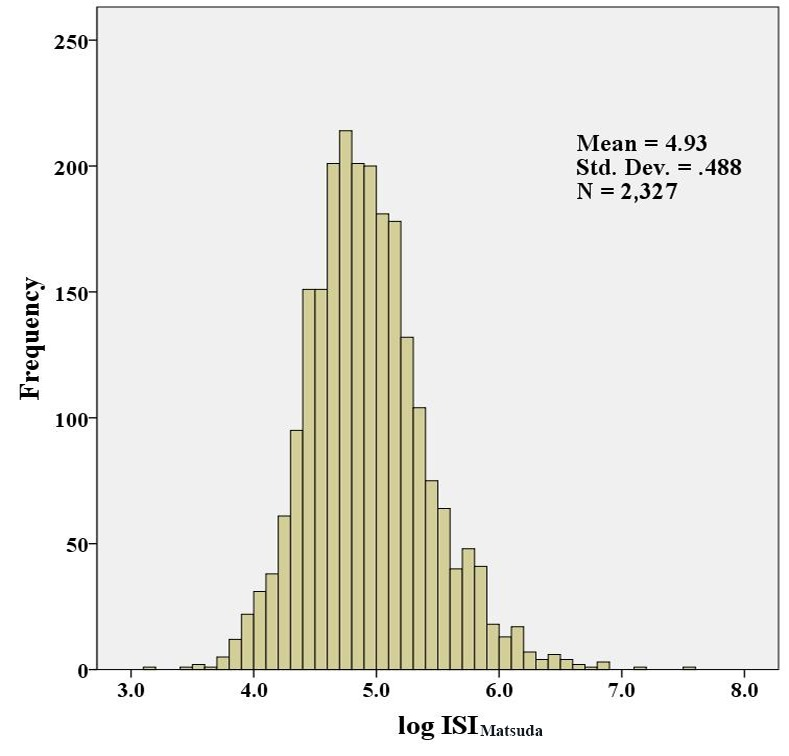

Supplement: S2 Fig — AUCins/glu: ratio of total area-under-the-insulin-curve to area-under-the-glucose-curve. (TIF) [file pone.0162204.s003.tif]

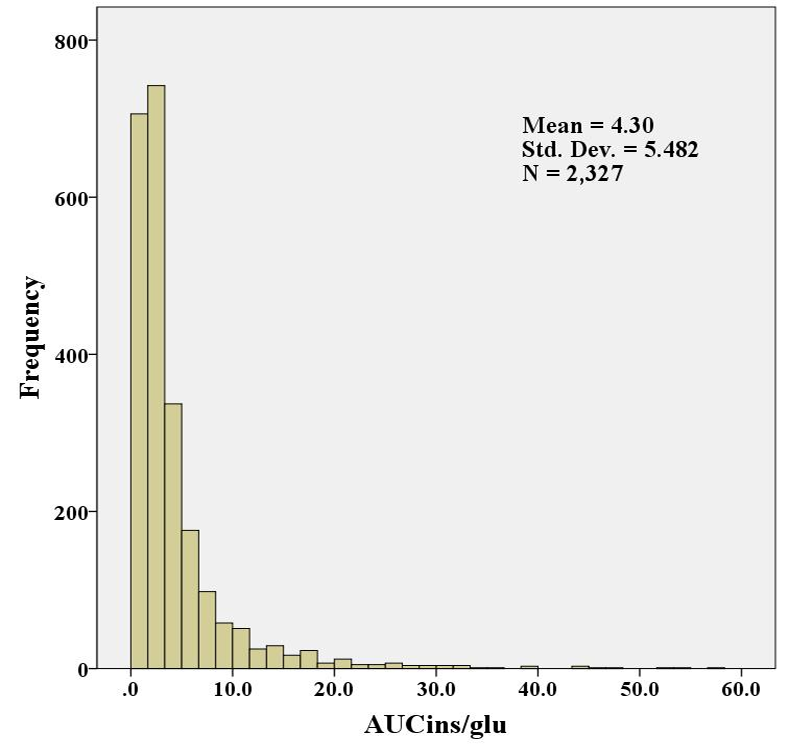

Supplement: S3 Fig — ISSI-2: Insulin Secretion-Sensitivity Index-2. (TIF) [file pone.0162204.s004.tif]

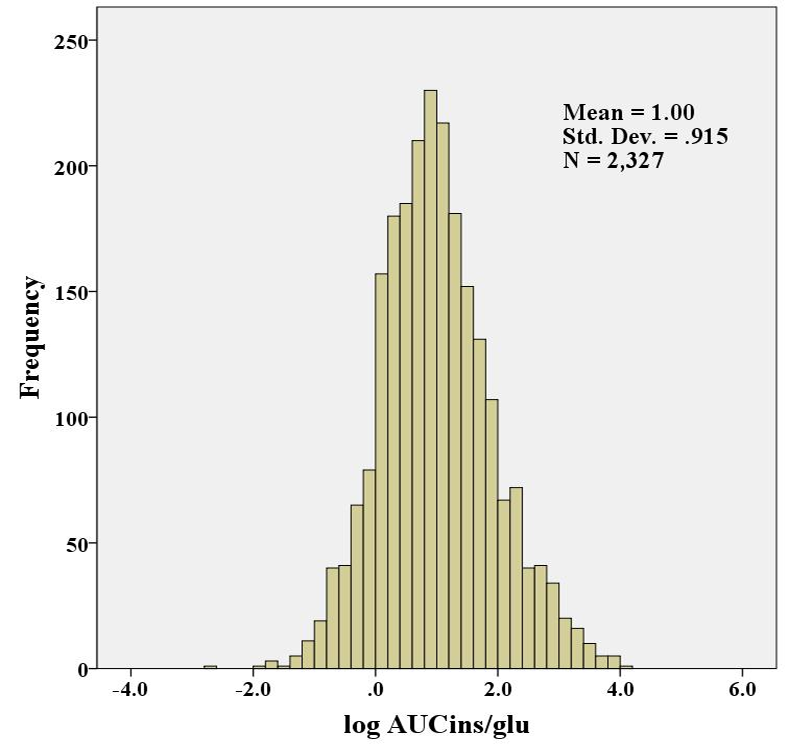

Supplement: S4 Fig — ISIMatsuda: insulin sensitivity index of Matsuda. (TIF) [file pone.0162204.s005.tif]

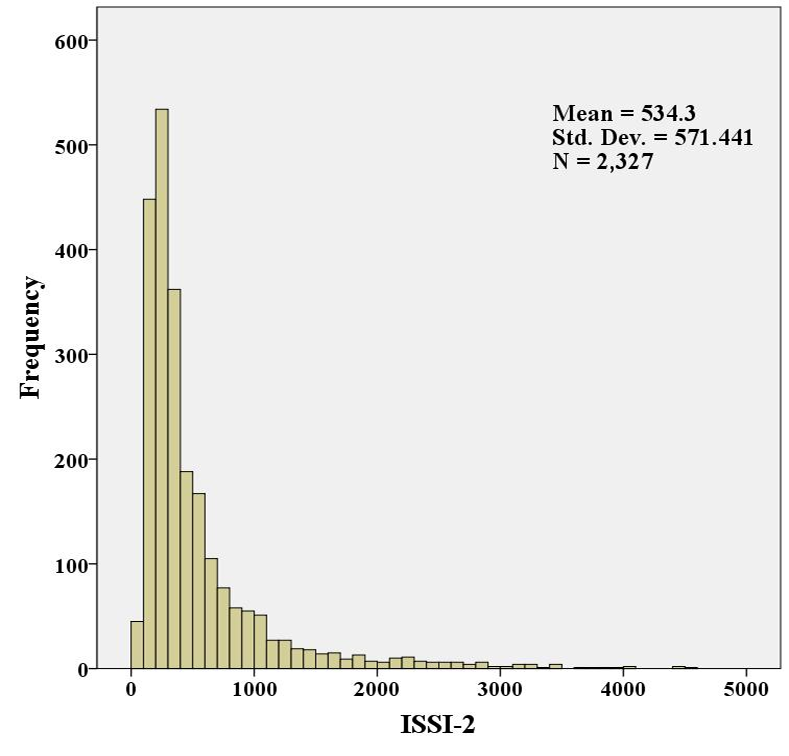

Supplement: S5 Fig — AUCins/glu: ratio of total area-under-the-insulin-curve to area-under-the-glucose-curve. (TIF) [file pone.0162204.s006.tif]

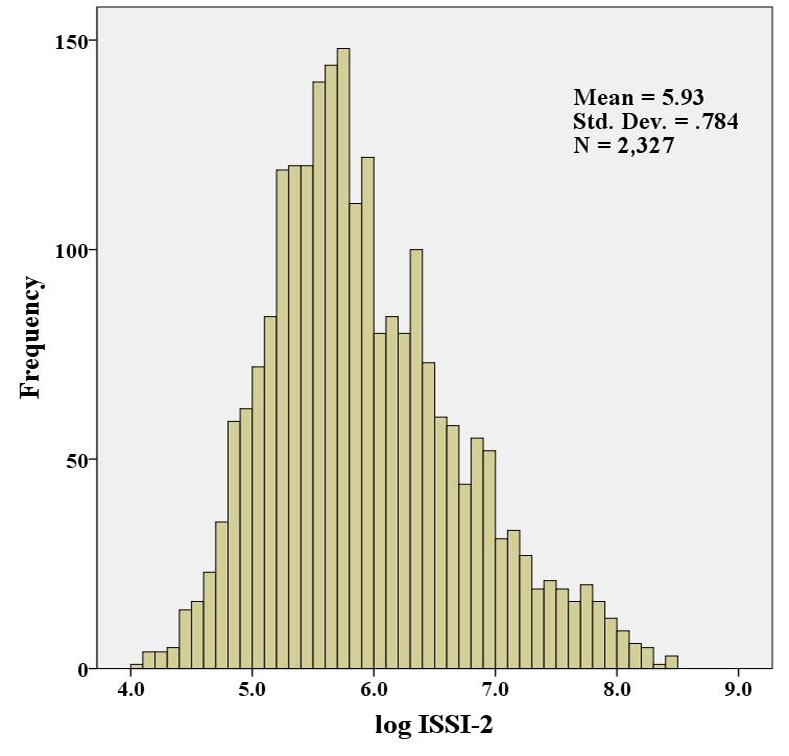

Supplement: S6 Fig — ISSI-2: Insulin Secretion-Sensitivity Index-2. (TIF) [file pone.0162204.s007.tif]
